# The Clinical Features of Cystic Parathyroid Adenoma in Chinese Population: A Single-Center Experience

**DOI:** 10.1155/2018/3745239

**Published:** 2018-07-15

**Authors:** Ya Hu, Ming Cui, Yu Xia, Zhe Su, Xiang Zhang, Quan Liao, Yuxin Jiang, Yupei Zhao

**Affiliations:** ^1^Department of General Surgery, Peking Union Medical College Hospital, Chinese Academy of Medical Sciences & Peking Union Medical College, Beijing 100730, China; ^2^Department of Ultrasound, Peking Union Medical College Hospital, Chinese Academy of Medical Sciences & Peking Union Medical College, Beijing 100730, China

## Abstract

**Objective:**

Cystic parathyroid adenoma is a rare cause of primary hyperparathyroidism, but its preoperative diagnosis and management remain inconclusive.

**Method:**

We retrospectively identified patients with cystic parathyroid adenomas who underwent surgery at Peking Union Medical College Hospital.

**Results:**

Patients with cystic parathyroid adenomas had higher serum intact parathyroid hormone and calcium levels, larger maximum tumor diameter, and lower serum inorganic phosphorus level than did those with solid adenomas. Patients with cystic adenomas were predominantly male, and hypercalcemic crisis and atypical adenomas were common. The accuracy of preoperative localization methodologies was lower in patients with cystic adenomas than in patients with solid adenomas. US-guided fine-needle aspiration was performed in 11 patients. In all patients, the iPTH level in cystic fluid was much higher than that in serum. No sign of recurrence was observed after a median follow-up of 39 months.

**Conclusions:**

Cystic parathyroid adenomas may not be as rare as previously reported. FNA may be a safe and feasible localization methodology for patients with inconclusive preoperative localization methodologies. Close follow-up is necessary for patients with cystic parathyroid adenomas, which account for a substantial proportion of atypical adenoma cases.

## 1. Introduction

Primary hyperparathyroidism (PHPT) is the most common disease in parathyroid glands and is accompanied by excessive secretion of parathyroid hormone (PTH) [1]. Single parathyroid adenomas account for approximately 80–90% of PHPT cases. The vast majority of parathyroid lesions are solid, but a few of these lesions can be cystic or partly cystic. Cystic parathyroid adenomas are reported to be very rare and account for 1-2% of PHPT cases [2]. Fewer than 300 cases have been reported in the literature, and most of these are case reports [3–5].

It is reported that cystic parathyroid adenomas account for approximately 9% of parathyroid cysts and are more common in males [6]. Cystic parathyroid adenomas are also known as functional parathyroid cysts, which are believed to be the result of cystic degeneration of existing parathyroid adenomas [7, 8]. Therefore, the fluid in most functional parathyroid cysts is not clear but bloody or dark brown in color [4, 9]. By contrast, 91% of parathyroid cysts are nonfunctional with normal serum PTH levels and are predominant in females [10]. Several explanations have been suggested for the pathogenesis of cystic parathyroid adenomas, including the retention of glandular secretion or the persistence of embryological remnants of the 3rd and 4th pharyngeal pouches [10].

Various nomenclatures have been used to describe cystic parathyroid adenomas, including symptomatic parathyroid cysts and hyperfunctioning cystic parathyroid glands. Furthermore, different arbitrary definitions have been used in previous studies to describe these lesions, such as predominant cystic lesions (>50% of the estimated volume of parathyroid lesions), cystic portions with diameter > 1 cm, and lesions with any cystic portion [8, 11, 12].

The rarity of cystic parathyroid lesions with PHPT may confound its diagnosis and treatment. These lesions may present as a neck mass, which are frequently mistaken as cystic thyroid lesions, especially in the presence of concomitant thyroid nodules. The accuracy of preoperative imaging modalities, such as neck ultrasound (US) scan and technetium-99m- (^99m^Tc-) methoxyisobutylisonitrile (MIBI) scintigraphy, has been reported to be dramatically reduced for patients with cystic parathyroid adenomas [13]. Nevertheless, a precise preoperative diagnosis of cystic adenomas can be followed with a minimally invasive parathyroidectomy instead of an extensive neck exploration procedure, which is very important for patients with severe PHPT and multisystem complications. Furthermore, the precise identification of the location of cystic adenomas is critical for optimizing the surgical approach, which may decrease the risk of cyst rupture and subsequent parathyromatosis. The use of fine-needle aspiration (FNA) biopsy has been discouraged for solid parathyroid lesions because of the risk of seeding along the needle tract or severe fibrosis complicating surgery [14–16]. However, FNA has been frequently used in the preoperative diagnosis of cystic adenomas according to many case reports. Although the primary results have been promising, the safety of FNA for this rare pathological condition needs further verification.

Due to its rarity, the clinical features of cystic and solid parathyroid adenomas have been compared seldom in published studies, and most clinical outcomes have been derived from case reports with limited follow-up durations [11]. There is a lack of universal consensus on the diagnosis and management of cystic parathyroid adenomas. Thus, there is a need for a better understanding of the characteristics and natural history of cystic parathyroid adenomas. Here, we presented a cohort of 37 patients with predominantly cystic lesions, which may represent the largest case series in recent years. The main purpose of the present study was to reassess the differences in clinical and biochemical features between cystic parathyroid adenomas and solid parathyroid adenomas. In addition, we evaluated the accuracy and safety of US-guided FNA for cystic parathyroid adenomas in this study.

## 2. Subjects and Methods

This is a retrospective review of a prospectively collected database that includes data from patients who were diagnosed with PHPT at Peking Union Medical College Hospital, a tertiary reference center in China. From January 2009 to March 2017, 907 consecutive patients underwent parathyroidectomy and were diagnosed with parathyroid adenomas on pathology. Cases with a histological diagnosis of parathyroid hyperplasia or carcinoma were not included. This study was approved by the Ethics Committee of Peking Union Medical College Hospital, and written informed consent was obtained from the patients for the use of their material for research purposes. The diagnosis of PHPT was based on the preoperative clinical and biochemical evidence of hyperparathyroidism, histological confirmation of the resected parathyroid tissue, and postoperative remission of hypercalcemia. Data including sex, age, clinical presentation, biochemical lab results, and preoperative localization analysis results were collected from the records of these patients. Cervical US scanning was performed for all patients before surgery by two experts in the field of parathyroid diseases. Before surgery, the diameters of parathyroid adenomas were estimated according to three-dimensional measurements. A planar MIBI scintigraphy was performed for all patients before surgery, except for patients who needed emergent parathyroidectomy or patients who were pregnant. The MIBI scan was considered positive for parathyroid lesions when a persistent focal uptake was detected 2 hours after the administration of MIBI. The diagnostic accuracy of these imaging modalities was assessed by comparing preoperative imaging data, intraoperative findings, and postoperative histopathologic results. Only the imaging results that could predict the exact location of parathyroid adenomas were considered true positives.

For patients whose neck US and/or MIBI scan results were inconclusive, an US-guided FNA was performed before the operation under local anesthesia. The intact PTH (iPTH) level in the aspirated cystic fluid was measured, and the aspirated cell cluster was cytopathologically examined.

All patients in this study underwent surgical intervention. Minimally invasive parathyroidectomy was performed in 721 patients under cervical plexus blocking, and the remaining patients underwent neck exploration under general anesthesia. The follow-up information was collected through a review of outpatient records and telephone interviews.

Based on sonographic and pathological evaluations, parathyroid adenomas were divided arbitrarily into two groups: cystic adenomas and solid adenomas. Cystic adenomas were defined as parathyroid adenomas with predominantly cystic lesions (>50% of the estimated volume of parathyroid lesions) [8, 13]. Adenomas with small cystic elements were categorized as solid adenomas, because their features on US scanning and MIBI scintigraphy were thus less likely to confound the preoperative diagnosis [13].

The macroscopic and histopathological features of all resected specimens were evaluated. The diagnosis of atypical adenoma was based on the presence of histological characteristics such as broad fibrous bands, trabecular growth, cytological atypia, marked mitosis, and the exclusion criteria corresponding to carcinomas [17, 18].

Continuous variables were described as the mean ± standard deviation (SD), and discrete data were reported as numbers or corresponding percentages. The nonparametric Mann–Whitney *U* test was used for intergroup comparisons of continuous variables, while categorical data were compared using two-tailed Fisher's exact test. *P* < 0.05 was considered statistically significant. SPSS version 22.0 for Windows (IBM SPSS, Chicago, Illinois, USA) was used for all analyses.

## 3. Results

**Figure 1:**
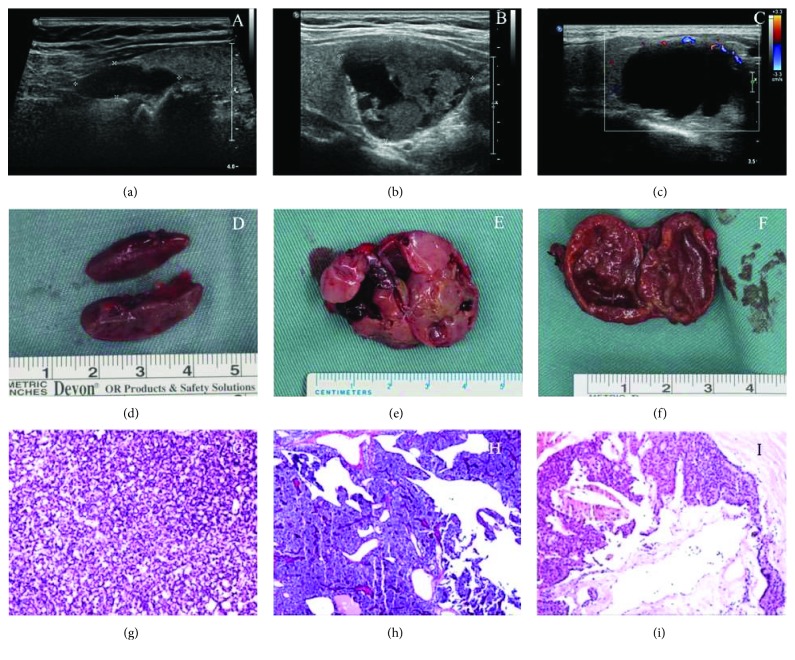
Sagittal ultrasound images (a, b, c), photographs (d, e, f), and histological features (hematoxylin/eosin staining) (g, h, i) of representative parathyroid adenomas. (a, d, g) Solid parathyroid adenoma without any cystic component. (b, e, h) Solid parathyroid adenoma with an insignificant cystic component. (c, f, i) Cystic parathyroid adenoma.

A total of 907 patients were enrolled in this study, among whom 37 had cystic parathyroid adenomas (16 males and 21 females) (Figures 1(c), 1(f), and 1(i)). The remaining 870 patients had solid adenomas (Figures 1(a), 1(d), and 1(g)), including 82 patients with microcystic adenomas (Figures 1(b), 1(e), and 1(h)). The comparison of clinicopathologic features between these two groups is presented in Table 1. The percentage of males was much higher in the cystic adenoma group than in the solid adenoma group (43.2% versus 25.6%, *P* = 0.022). More patients presented with hypercalcemic crisis in the cystic adenoma group than in the solid adenoma group. The incidence of urolithiasis, acute pancreatitis, and bone fracture was similar between the two groups. The preoperative serum levels of total calcium and iPTH were significantly higher in patients with cystic adenomas than in patients with solid adenomas. The level of inorganic phosphorus was lower in patients with cystic adenomas than in patients with solid adenomas. No significant differences were observed in the serum level of creatinine or alkaline phosphatase (ALP).

In this cohort, neck US scanning was able to identify cystic lesions in 36 patients (97.3%) in the cystic adenoma group; however, 10 of these cases were misdiagnosed as having thyroid cysts or other neck lesions. Conclusive US imaging results were obtained for 26 patients (70.3%), a percentage that was much lower than that for patients with solid adenomas (96.8%, *P* = 0.001). Meanwhile, the difference in the accuracy of MIBI scintigraphy between the two groups was also significant. MIBI scintigraphy was negative in 19.4% patients with cystic adenomas, which was markedly higher than that in patients with solid adenomas (4.3%, *P* = 0.001). The maximum diameter of adenomas in the cystic adenoma group was much larger than that in the solid adenoma group (40.5 ± 13.9 mm versus 21.6 ± 9.6 mm, *P* = 0.001).

US-guided FNA was performed in 11 patients (30%) for whom the results of MIBI scintigraphy and/or US imaging were inconclusive. In all cases, the level of iPTH in the obtained cystic fluid was more than 2500 pg/mL, which was markedly higher than the serum level of iPTH measured at the same time. The cytological findings of aspirated specimens were helpful for the preoperative diagnosis of parathyroid lesions in 4 patients (36.4%). Parathyroid tumor cells were found in one patient, and small numbers of parathyroid cells were identified in three other patients.

The histopathologic evaluation of all resected cystic parathyroid adenomas revealed cystic structures that were primarily surrounded by hyperplastic parathyroid tissues. Compared with those in the solid adenoma group, more atypical adenomas were identified in the cystic adenoma group (5.4% versus 24.3%, *P* = 0.001). Furthermore, cystic adenomas were not associated with multiple endocrine neoplasia type 1 (MEN-1), multiglandular disease, or ectopic mediastinal parathyroid adenomas.

For all patients with cystic adenomas, the serum iPTH level on postoperative day 1 decreased to a value within the normal range. Follow-up information was available for 35 patients, with a median follow-up duration of 30 months (range, 8–103 months). The symptoms of these patients markedly improved, and the serum calcium level remained with the normal range. Moreover, for the 11 patients who underwent US-guided FNA, PHPT was biochemically cured, as determined by normal serum calcium and iPTH levels over a median follow-up duration of 39 months (range, 8–84 months).

## 4. Discussion

Cystic parathyroid adenomas are traditionally believed to represent a small percentage of PHPT cases. However, the incidence of cystic adenomas was 4.1% in the present study, which is much higher than the incidence (<3%) in most previous reports from western countries [8, 19]. Another study from Iran reported a much higher incidence of cystic parathyroid adenoma (17%) [11]. One of the reasons for this inconsistency may be the differences in the criteria applied for patient selection. More patients with severe PHPT were enrolled in the studies from developing countries with limited health services. Unlike patients in western countries who frequently present with minor symptoms of PHPT based on biochemical screening, most patients in China and other developing countries are more likely to present with obvious symptoms and extreme hypercalcemia, which are accompanied with larger adenomas [20]. Furthermore, large tumors are common for cystic parathyroid adenomas, which may partly explain the discrepancies described above [8, 13].

In agreement with previous studies, cystic parathyroid adenomas in the present cohort were found to be associated with high serum levels of iPTH and calcium [5, 21]. Additionally, a higher incidence of hypercalcemic crisis was observed in patients with cystic adenomas than in patients with solid adenomas. The rupture of cystic parathyroid adenomas has been reported to release a large amount of PTH into the blood circulation, a phenomenon that can cause parathyromatosis and hematosis [22, 23]. Fortunately, none of the patients in our cohort suffered from this dangerous event. Theoretically, other symptoms associated with severe PHPT can be expected to be observed more frequently in patients with cystic adenomas. However, the incidence of urolithiasis, acute pancreatitis, and bone fracture was similar in the two groups in our study. This inconsistency may be explained by the notion that cystic adenomas arise from acute necrosis and hemorrhage in parathyroid adenomas within a relatively short time, which results in the release of large amounts of PTH into the circulation [24]. An abrupt rise in serum PTH may lead to a significant increase in serum calcium levels and trigger a hypercalcemic crisis; however, urolithiasis and osteoporosis cannot develop within a limited time.

The preoperative diagnosis of cystic parathyroid adenomas remains challenging. Even though the combined use of US scanning and MIBI scintigraphy may identify most solid parathyroid adenomas, their accuracy is significantly low for cystic adenomas, especially in the presence of concomitant thyroid nodules [13, 25]. In the present cohort, the accuracy of neck US scanning is dramatically low, even in the hands of expert (70.3% in cystic adenomas versus 96.8% in solid adenomas). Cystic parathyroid adenomas around the thyroid may be frequently misinterpreted as cystic thyroid lesions, as the classic sonographic features of parathyroid lesions, such as hypoechoic nodules with abundant blood flow, are absent in most cystic parathyroid adenomas. In addition, there may be no uptake or dubious marginal uptake of MIBI in cystic parathyroid adenomas, which can make preoperative diagnosis even more difficult [26, 27]. In our study, the accuracy of planar MIBI scintigraphy was 80.6% in the cystic adenoma group, which was much lower than that in the solid adenoma group (95.7%). These findings are in line with the results of previous studies [13].

The use of FNA is discouraged in the diagnosis of parathyroid lesions because of the risk of seeding along the needle tract [14, 28]. However, it is believed to be a useful diagnostic method for cystic parathyroid adenoma [13, 19, 29]. The level of iPTH in the aspirated cystic fluid was much higher than that in the serum, although thyroid cystic fluid is known to contain high levels of thyroglobulin [30–32]. In this study, the sensitivity and specificity of FNA were both 100% for all patients. Furthermore, no evidence of recurrence was observed during the follow-up in this group of patients. By contrast, the results of the cytological examination of the aspirated specimen were helpful for only a small proportion of patients (36.4%). Therefore, the detection of PTH in the aspiration fluid seems to be promising and convenient. Nevertheless, the use of FNA for patients with cystic adenomas should still be regarded with caution before completion of further studies to assess the safety with long-term follow-up.

Until now, few studies have focused on the pathology of cystic parathyroid adenomas, and these lesions have been generally thought to be secondary to degenerative changes in parathyroid tumors [33]. Even though atypical adenomas have been seldom identified in cystic parathyroid adenomas in previous case reports, for the first time, we found that cystic parathyroid adenomas are closely related to atypical adenomas [9, 34]. Approximately one-fourth of the resected cystic adenomas in the present cohort were identified as atypical parathyroid adenomas upon pathological examination. The WHO histological criteria for parathyroid carcinoma include unequivocal evidence of invasion into the adjacent tissue, vascular or perineural permeation, and the presence of metastases. Atypical parathyroid adenomas, which present with atypical features but do not fulfill the criteria of carcinoma, have been reported to have a low but real risk of recurrence [18]. Although the causal relationship between cystic changes and atypical adenoma is unknown, close follow-up is necessary for patients with these lesions.

There were several limitations in this study that warrant further discussion. This study was a retrospective, single-institution study, which may introduce selection bias. Another limitation of the present study was the relatively small cohort size of patients with cystic parathyroid adenomas, even though this is one of the largest published series. In addition, the follow-up time for patients who underwent FNA was limited. Thus, the safety of FNA for cystic parathyroid adenomas needs to be further verified.

In conclusion, cystic parathyroid adenomas may not be as rare as previously reported, especially in severe PHPT. Cystic parathyroid adenomas are associated with large lesions, high serum levels of iPTH and calcium, a high risk of hypercalcemic crisis, and low accuracy of preoperative localization examinations. FNA may be a safe and feasible localization method for cystic parathyroid adenomas. Close follow-up is necessary for patients with cystic parathyroid adenomas, which account for a substantial percentage of atypical parathyroid adenoma cases.

## Figures and Tables

**Table 1:** Comparison of the clinicopathological features between patients with solid parathyroid adenomas and patients with cystic parathyroid adenomas.

Feature	Solid parathyroid adenomas (*n* = 870)	Cystic parathyroid adenomas (*n* = 37)	*P* value
Male-to-female ratio	1 : 2.9	1 : 1.3	**0.022** ^∗^
Age at diagnosis (years)	52.8 ± 13.9	54. 6 ± 14.2	0.417
Serum iPTH level (pg/mL)	408.7 ± 503.9	620.0 ± 501.3	**0.001** ^∗∗^
Serum calcium level at diagnosis (mmol/L)	2.89 ± 0.35	3.13 ± 0.48	**0.002** ^∗∗^
Serum inorganic phosphorus (mmol/L)	0.84 ± 0.19	0.73 ± 0.22	**0.001** ^∗∗^
Alkaline phosphatase (U/L)	207.9 ± 385.5	209.6 ± 325.0	0.224
Serum creatinine (*μ*mol/L)	68.8 ± 28.7	93.2 ± 114.1	0.137
Hypercalcemic crisis (%)	4.4	13.5	**0.027** ^∗^
Urolithiasis (%)	34.7	51.4	0.052
Acute pancreatitis (%)	2.2	5.4	0.210
Bone fracture (%)	15.4	13.5	1.000
Multiglandular disease (%)	4.5	8.1	0.242
MEN (%)	4.5	2.7	1.000
Accuracy of US scanning (%)	96.8	70.3	**0.001** ^∗∗^
Accuracy of MIBI scintigraphy (%)	95.7	80.6	**0.001** ^∗∗^
Mediastinal ectopic parathyroid adenoma (%)	1.3	2.7	0.395
Maximum tumor diameter (mm)	21.6 ± 9.6	40.5 ± 13.9	**0.001** ^∗∗^
Atypical adenoma (%)	5.4	24.3	**0.001** ^∗∗^

Asterisks (∗) indicate statistically significant differences between the two groups (^∗^*P* < 0.05 and ^∗∗^*P* < 0.01). iPTH: intact parathyroid hormone; MEN: multiple endocrine neoplasia.

## Data Availability

All related clinical data in this manuscript are stored and can be accessed in our clinical database of hyperparathyroidism.
